# Misadventures in Toxicology: Concentration Matters for Testosterone-Induced Neurotoxicity

**DOI:** 10.3390/toxics11030258

**Published:** 2023-03-10

**Authors:** Cyril Willson

**Affiliations:** EuSci LLC, 1309 S 204th St, #293, Elkhorn, NE 68022, USA; cmwillson@gmail.com

**Keywords:** pharmacokinetics, testosterone, neurotoxicity, blood–brain barrier

## Abstract

Testosterone is the predominant androgen in men and has important physiological functions. Due to declining testosterone levels from a variety of causes, testosterone replacement therapy (TRT) is increasingly utilized, while testosterone is also abused for aesthetic and performance-enhancing purposes. It has been increasingly speculated that aside from more well-established side effects, testosterone may cause neurological damage. However, the in vitro data utilized to support such claims is limited due to the high concentrations used, lack of consideration of tissue distribution, and species differences in sensitivity to testosterone. In most cases, the concentrations studied in vitro are unlikely to be reached in the human brain. Observational data in humans concerning the potential for deleterious changes in brain structure and function are limited by their inherent design as well as significant potential confounders. More research is needed as the currently available data are limited; however, what is available provides rather weak evidence to suggest that testosterone use or abuse has neurotoxic potential in humans.

## 1. Introduction

Testosterone is the predominant androgen in men and serves important physiological roles in both men and women [1]. In healthy eugonadal men, testosterone is produced primarily by the Leydig cells of the testicles and results in the total daily production of approximately 6–7 mg/day, although a range of 3–10 mg total testosterone production is often cited as well [1,2,3,4,5,6,7,8,9,10]. This production rate results in the often cited “normal range” for total testosterone in males of between 300–1000 ng/dL in plasma, although this range varies based upon the laboratory and population examined [11,12].

Testosterone levels may generally decline with advancing age and may eventually reach a point of “testosterone deficiency,” although there are other causes of low testosterone, including certain injuries, medications, obesity, illnesses, radiation exposure, and genetic conditions [13,14,15,16,17]. This deficiency is characterized most often by symptoms and testosterone levels (e.g., generally total testosterone below 300 ng/dL, although other parameters may be used) [13,14,15,16,17]. The treatment for testosterone deficiency, often referred to as “testosterone replacement therapy” or TRT, is designed to decrease the symptoms of low testosterone, such as decreased libido, sexual function, and lean body mass, while ideally maintaining testosterone levels within the normal range [13,14,15,16,17].

Testosterone, while produced endogenously by humans, is still (in the most technical sense) an anabolic-androgenic steroid (AAS), albeit one that is endogenously produced. While TRT is generally considered to be a rather safe practice, there are of course risks for adverse effects (e.g., erythrocytosis and acne) as with any pharmacotherapy [13,14,15,16,17]. While the adverse effects of testosterone—and in a broader sense, all anabolic-androgenic steroids—are fairly well described, more recent attention has been devoted to potential neurological side effects [18,19]. While short-term alterations in psychiatric and cognitive variables have been noted with acute administration in men, these changes are primarily thought to involve neurochemical changes or alterations in signaling pathways leading to temporary alterations in function rather than permanent neurological changes [20,21,22,23,24].

Relatedly, it has also been proposed that testosterone, especially in the “high normal” and “supraphysiological” range, may cause neurotoxicity, resulting in an increased risk for neurodegenerative diseases (e.g., Parkinson’s, Alzheimer’s, Huntington’s) or at the very least general cognitive decline, especially with chronic use, noting that these, like many of the adverse effects of androgens in general are dose and duration dependent [18,19,25,26,27,28]. Such a hypothesis requires a more extensive examination into the evidence cited for such claims.

## 2. In Vitro Data

### 2.1. Concentration Matters

Several groups have investigated the potential neurotoxicity of testosterone in vitro by utilizing human and rodent cell lines [25,26,27,28]. Authors of these studies have proposed that concentrations as low as 100 nmol of testosterone may be neurotoxic, while also indicating that this concentration is consistent with the “high normal range” of total blood testosterone in men, and acknowledging that concentrations of 1 µmol or greater are in the supraphysiological range [26].

First, it should be noted that contrary to the claims of some authors [26], 100 nmol is not within any accepted physiological range for eugonadal males. In fact, it is nearly 3 times greater than the upper limit of normal for total blood testosterone levels in eugonadal males [12,13,14]. The typical replacement doses utilized for TRT would not be expected to reach this concentration on average (see Table 1) [29,30,31,32,33,34,35,36,37,38,39,40,41,42,43,44,45,46]. Furthermore, even amongst those that are abusing testosterone, consistent concentrations of this magnitude in plasma are not expected, except for those using quantities of 500–600 mg or more of testosterone cypionate/enanthate weekly (see Table 1) [30,31,32,33,34,35,36,37,38,39,40,41,42,43,44,45,46].

While pharmacokinetic data gathered in humans administered varying doses of testosterone esters either intramuscularly or subcutaneously vary considerably depending upon the route of administration and sampling method, these data generally show that only quantities used as part of testosterone abuse are capable of reaching concentrations of 100 nmol or greater on a consistent basis. Other authors have indicated that concentrations of 100–500 µmol are typically reached with a supraphysiological dose of 600 mg of testosterone enanthate weekly [27], which is also incorrect (see Table 1). This same group’s work was used in lay press articles to claim that even levels of testosterone seen with TRT can “lead to a catastrophic loss of brain cells” [47]. Concentrations well beyond 100 nmol, and especially into the 1 µmol to 100 µmol range, for total testosterone are highly unlikely, even in those abusing testosterone for athletic/aesthetic purposes. These concentrations have not been reached in studies utilizing supraphysiological doses of exogenous testosterone (see Table 1). Furthermore, the only documented case of testosterone overdose in the literature was in a young man who experienced a cerebrovascular accident with a total plasma testosterone concentration of 395 nmol (11,400 ng/dL) [48].

It should also be noted that these concentrations discussed relate to total testosterone (i.e., testosterone that is unbound and bound by sex hormone binding globulin (SHBG) and albumin), while “free testosterone” or testosterone that is not bound to SHBG and albumin constitutes only 2–4% of total circulating testosterone; 50–60% of total testosterone is bound by SHBG and is generally not considered available to tissues such as the brain [13,14,16,17]. Thus, the utilization of high concentrations of free testosterone in vitro results in an untenable comparison with total plasma testosterone concentrations reached even after supraphysiological doses of exogenous testosterone. While it can certainly be argued that the relationship between testosterone and SHBG is dynamic and can be altered in cases of exogenous administration (amongst other variables), ultimately what matters most is the available concentration in brain tissue (see Section 2.2).

It is also important to note that even in cases where a given concentration has been shown to have toxic effects in a given cell line (i.e., ≥100 nmol), maintaining blood concentrations of this magnitude would also be necessary, as short-term exposure (e.g., 24 h) in vitro has not been shown to be capable of producing cellular damage [26].

Thus, in this instance the concentrations used in vitro are not reflective of what is seen in vivo in humans. Just as with pharmacological targets, utilizing extreme concentrations may not accurately reflect the actual risk of cell/tissue damage [49,50].

### 2.2. Consideration of Tissue Distribution

While doses of exogenous testosterone normally utilized for TRT purposes are unlikely to elevate plasma testosterone to concentrations that have been shown to have neurotoxic potential in vitro, it is even more unlikely once tissue distribution is considered. In this instance, it is known that blood concentrations of testosterone overestimate the levels found in the human brain by 3–10 fold [51,52,53,54]. Specifically, brain tissue concentrations are typically around 1 ng/g of tissue on average. This is likely explained, at least in part, by the restriction of SHBG-bound testosterone to blood and its inability to cross the blood–brain barrier, as well as the local metabolism of testosterone [54]. While it is questionable whether exogenous testosterone administration could result in a substantially disproportionate amount of testosterone to distribute to the brain, the available evidence (albeit limited), utilizing cerebrospinal fluid (CSF) as a surrogate for levels in the brain relative to plasma, suggests that the brain maintains a relative equilibrium with the blood and that any perturbations are rapidly corrected to maintain this relationship [55,56,57,58,59,60,61].

Conversely, the synthetic 17-alpha alkylated derivative of testosterone [62], methyltestosterone, has been shown to substantially favor the CSF over blood levels. However, this likely reflects increased blood–brain barrier (BBB) penetration, presumably due to greater lipophilicity and reduced binding to SHBG. These differences are likely due to the reduced hydrogen bonding of the 17-β-hydroxyl group of testosterone due to steric hindrance afforded by the bulky methyl substituent at the 17-α position [63,64]. Certainly, such seemingly small chemical and physicochemical differences between methyltestosterone and testosterone may not be a complete explanation for such differences, but an established equilibrium for a molecule that has evolved with mammals for millions of years versus a synthetically altered version is not completely surprising. It should also be noted that even in this study, though often cited as evidence that AASs are capable of reaching micromolar concentrations in the human brain [28], mean concentrations were actually 233 nmol, with a maximum range of 898 nmol in the CSF, which itself can only be considered a potential surrogate of brain concentrations [58,65].

### 2.3. Species Differences in Sensitivity

Aside from the limitations discussed previously, in some instances, rodent cell lines were used for in vitro assays evaluating the potential for neurotoxicity. The issue with such use is the question of whether the chosen species possesses the same sensitivity as humans [66]. It is unknown in this case, but there are instances of other tissue types (e.g., liver) demonstrating that humans are less sensitive than rodents with respect to the cellular toxicity of testosterone [67]. In addition, data show that other molecules may have substantially divergent neurotoxic potential in humans as compared to rodents [66]. It is interesting to note that while a direct comparison is not possible, the only study to use human neuronal cells demonstrated toxicity only at 1 µmol [27], while the rat-derived N27 cells demonstrated toxicity at 100 nmol [26].

Aside from the potential interspecies (as well as different cell types from the same species) differences in the sensitivity of different cell types, it must also be considered whether their metabolic capabilities accurately reflect those seen in normal humans; whether steroid receptor content is comparable between cell types; and even if the same cell line may have divergent properties from the original after repeated passage [66,68,69,70,71].

## 3. Observational Studies

While beyond the scope of this opinion paper, it is worth noting that several groups have reported findings indicating that AAS users may suffer from brain alterations and cognitive dysfunction [19,72,73,74,75]. However, these study designs generally do not allow for a causal relationship to be established. Furthermore, perhaps more importantly, these studies are vulnerable to major confounders, including the known polypharmacy (including other licit and illicit drugs of abuse) that anabolic steroid users self-administer; the reliability of self-reported data and potential reverse causation considering the role of pre-existing factors, such as addiction/substance abuse/dependence predisposition; psychiatric and psychological conditions; and lower IQ increasing the likelihood of AAS-dependence [76,77,78,79,80,81,82,83,84,85,86,87,88]. Certainly, those that may be using or even abusing testosterone may wish to be informed of potential serious adverse effects. However, by focusing on the potential neurodegenerative disease due to androgen use, especially in light of the limited data to support the notion of such a hazard, it could be argued that this risks further alienating a population that already views the opinions of physicians and mainstream medical advice with some skepticism [89]. It was not long ago that those abusing androgens were told that they were not actually effective [90], while the risks of their use and abuse may have been exaggerated [91,92,93,94,95]. Nevertheless, it should be acknowledged that potential risks with long-term abuse exist, especially with respect to adverse cardiovascular effects [96], while the health effects associated with long-term TRT use are still being investigated [97].

## 4. Implications

While testosterone use for TRT is still subject to some controversy, the available data are rather weak to suggest that neurological damage or an increased risk of neurodegenerative disease is a risk with long-term use either at therapeutic doses or those generally used for athletic/aesthetic purposes. In vitro data utilizing concentrations that are irrelevant to in vivo administration should not be relied upon as supportive evidence of neurological damage. More research is needed to determine if long-term androgen use/abuse is a risk factor for neurological damage or neurodegenerative disease.

## Figures and Tables

**Table 1:** Comparison of in vivo testosterone plasma concentrations with neurotoxic in vitro concentrations.

Testosterone Preparation	Dose (mg)	Route of Administration	Single or Multi-Dose	Mean Plasma Concentrations in nmol (ng/dL)	Concentration in Vitro Demonstrating Neurotoxicity in nmol (ng/dL)	Cell Line Type (Species)	References
Testosterone Enanthate	250	Intramuscular	Single	39.4 (1136)	100 (2884)	N27 (rat)	[26,30]
Testosterone Enanthate	200	Intramuscular	Single	68.1 (1965)	*	GT1-7 (mouse)	[26,31]
Testosterone Enanthate	100	Intramuscular	Single	40.9 (1181)	1000–10,000 (28,843–288,428)	SH-SY5Y (human)	[27,31]
Testosterone Enanthate	200	Intramuscular	Multi (Bi-Weekly)	50.7 (1462)	1000–10,000 (28,843–288,428)	Pure Cortical Neurons (rat)	[25,32]
Testosterone Enanthate	100	Intramuscular	Multi (Weekly)	24.9 (718) (mean between injections)	1000 (28,843)	Mixed Cortical Cells (rat)	[25,33]
Testosterone Enanthate	300	Intramuscular	Multi (Weekly)	51.8 (1494)			[33]
Testosterone Enanthate	100	Subcutaneous	Multi (Weekly)	46.7 (1346 mean Cmax)	100,000 (2884,282)	Mixed Cortical Cells (rat)	[28,34]
Testosterone Enanthate	200	Intramuscular	Multi (Bi-Weekly)	78.4 (2262 mean Cmax; Range up to 167.8 (4840)			[34]
Testosterone Cypionate	250	Intramuscular	Multi (Weekly)	<52 (<1500)			[35]
Testosterone Cypionate	500	Intramuscular	Multi (Weekly)	<86.7 (<2500)			[35]
Testosterone Enanthate	200	Intramuscular	Multi (Weekly)	77.5 (2235)			[36]
Testosterone Enanthate	200	Intramuscular	Multi (Weekly)	38.4 (1108)			[37]
Testosterone Enanthate	600	Intramuscular	Multi (Weekly)	76.9 (2218 nadir)			[38]
Testosterone Enanthate	600	Intramuscular	Multi (Weekly)	98–112.5 (2828–3244)			[39]
Testosterone Enanthate	400	Intramuscular	Multi (Bi-Weekly)	39.7 (1146)			[40]
Testosterone Enanthate	600	Intramuscular	Multi (Weekly)	76.9 (2218 younger men)			[41]
-	-	-		124.9 (3603 older men)			[41]
Testosterone Enanthate	600	Intramuscular	Multi (Weekly)	92.0 (2654)			[42]
Testosterone Enanthate	600	Intramuscular	Multi (Weekly)	82.2 (2370 nadir)			[43]
Mixed Testosterone Esters (Sustanon 250)	250	Intramuscular	Single	71.0 (2048) Range up to 121.0 (3490)			[44]
Mixed Testosterone Esters (Sustanon 250)	250	Intramuscular	Single	81.4 (2348)			[45]
Testosterone Cypionate	200	Intramuscular	Single	38.6 (1112)			[46]

* No toxicity demonstrated.

## Data Availability

Not applicable.

## References

[1] Spritzer M.D., Roy E.A. (2020). Testosterone and Adult Neurogenesis. Biomolecules.

[2] Southren A.L., Gordon G.G., Tochimoto S. (1968). Further study of factors affecting the metabolic clearance rate of testosterone in man. J. Clin. Endocrinol. Metab..

[3] Horton R., Shinsako J., Forsham P.H. (1965). Testosterone production and metabolic clearance rates with volumes of distribution in normal adult men and women. Acta Endocrinol..

[4] Southren A.L., Tochimoto S., Carmody N.C., Isurugi K. (1965). Plasma production rates of testosterone in normal adult men and women and in patients with the syndrome of feminizing testes. J. Clin. Endocrinol. Metab..

[5] Southren A.L., Gordon G.G., Tochimoto S., Pinzon G., Lane D.R., Stypulkowski W. (1967). Mean plasma concentration, metabolic clearance and basal plasma production rates of testosterone in normal young men and women using a constant infusion procedure: Effect of time of day and plasma concentration on the metabolic clearance rate of testosterone. J. Clin. Endocrinol. Metab..

[6] Wang C., Catlin D.H., Starcevic B., Leung A., DiStefano E., Lucas G., Hull L., Swerdloff R.S. (2004). Testosterone metabolic clearance and production rates determined by stable isotope dilution/tandem mass spectrometry in normal men: Influence of ethnicity and age. J. Clin. Endocrinol. Metab..

[7] Hodgson Y., Hudson B. (1983). Leydig cell function. Monogr. Endocrinol..

[8] Mori H., Hiromoto N., Nakahara M., Shiraishi T. (1982). Stereological analysis of Leydig cell ultrastructure in aged humans. J. Clin. Endocrinol. Metab..

[9] Mori H., Motta P.M. (1984). Ultrastructure and stereological analysis of Leydig cells. Ultrastructure of Endocrine Cells and Tissues. Electron Microscopy in Biology and Medicine.

[10] Basaria S. (2014). Male hypogonadism. Lancet.

[11] Shoskes J.J., Wilson M.K., Spinner M.L. (2016). Pharmacology of testosterone replacement therapy preparations. Trans. Androl. Urol..

[12] Matsumoto A.M., Bremner W.J. (2004). Serum testosterone assays—Accuracy matters. J. Clin. Endocrinol. Metab..

[13] Dandona P., Rosenberg M.T. (2010). A practical guide to male hypogonadism in the primary care setting. Int. J. Clin. Pract..

[14] Bhasin S., Brito J.P., Cunningham G.R., Hayes F.J., Hodis H.N., Matsumoto A.M., Snyder P.J., Swerdloff R.S., Wu F.C., Yialamas M.A. (2018). Testosterone Therapy in Men with Hypogonadism: An Endocrine Society Clinical Practice Guideline. J. Clin. Endocrinol. Metab..

[15] Mulhall J.P., Trost L.W., Brannigan R.E., Kurtz E.G., Redmon J.B., Chiles K.A., Lightner D.J., Miner M.M., Murad M.H., Nelson C.J. (2018). Evaluation and Management of Testosterone Deficiency: AUA Guideline. J. Urol..

[16] Jayasena C.N., Anderson R.A., Llahana S., Barth J.H., MacKenzie F., Wilkes S., Smith N., Sooriakumaran P., Minhas S., Wu F.C.W. (2022). Society for Endocrinology guidelines for testosterone replacement therapy in male hypogonadism. Clin. Endocrinol..

[17] Park H.J., Ahn S.T., Moon D.G. (2019). Evolution of Guidelines for Testosterone Replacement Therapy. J. Clin. Med..

[18] Pomara C., Neri M., Bello S., Fiore C., Riezzo I., Turillazzi E. (2015). Neurotoxicity by synthetic androgen steroids: Oxidative stress, apoptosis, and neuropathology: A review. Curr. Neuropharmacol..

[19] de Azevedo Cruz Seara F., Fortunato R.S., Carvalho D., Nascimento J.H.M. (2017). Neurophysiological Repercussions of Anabolic Steroid Abuse: A Road into Neurodegenerative Disorders. Sex Hormones in Neurodegenerative Processes and Diseases.

[20] Kranz G.S., Spies M., Vraka C., Kaufmann U., Klebermass E.M., Handschuh P.A., Ozenil M., Murgaš M., Pichler V., Rischka L. (2021). High-dose testosterone treatment reduces monoamine oxidase A levels in the human brain: A preliminary report. Psychoneuroendocrinology.

[21] Spurny-Dworak B., Handschuh P., Spies M., Kaufmann U., Seiger R., Klöbl M., Konadu M.E., Reed M.B., Ritter V., Baldinger-Melich P. (2022). Effects of sex hormones on brain GABA and glutamate levels in a cis- and transgender cohort. Psychoneuroendocrinology.

[22] O’Connor D.B., Archer J., Hair W.M., Wu F.C. (2001). Activational effects of testosterone on cognitive function in men. Neuropsychologia.

[23] Carré J.M., Geniole S.N., Ortiz T.L., Bird B.M., Videto A., Bonin P.L. (2017). Exogenous Testosterone Rapidly Increases Aggressive Behavior in Dominant and Impulsive Men. Biol. Psychiatry.

[24] Foradori C.D., Weiser M.J., Handa R.J. (2008). Non-genomic actions of androgens. Front. Neuroendocrinol..

[25] Caraci F., Pistarà V., Corsaro A., Tomasello F., Giuffrida M.L., Sortino M.A., Nicoletti F., Copani A. (2011). Neurotoxic properties of the anabolic androgenic steroids nandrolone and methandrostenolone in primary neuronal cultures. J. Neurosci. Res..

[26] Cunningham R.L., Giuffrida A., Roberts J.L. (2009). Androgens induce dopaminergic neurotoxicity via caspase-3-dependent activation of protein kinase C-delta. Endocrinology.

[27] Estrada M., Varshney A., Ehrlich B.E. (2006). Elevated testosterone induces apoptosis in neuronal cells. J. Biol. Chem..

[28] Zelleroth S., Nylander E., Nyberg F., Grönbladh A., Hallberg M. (2019). Toxic Impact of Anabolic Androgenic Steroids in Primary Rat Cortical Cell Cultures. Neuroscience.

[29] El-Khatib F.M., Huynh L.M., Kopelevich A., Osman M.M., Choi E., Nguyen J.T., Dianatnejad S., Wu Q., Olivas M.G., Spitz A. (2022). Comparative assessment of outcomes and adverse effects using two different intramuscular testosterone therapy regimens: 100 mg IM weekly or 200 mg IM biweekly. Int. J. Impot. Res..

[30] Behre H.M., Nieschlag E., Nieschlag E., Behre H.M. (1998). Comparative pharmacokinetics of testosterone esters. Testosterone.

[31] Sokol R.Z., Palacios A., Campfield L.A., Saul C., Swerdloff R.S. (1982). Comparison of the kinetics of injectable testosterone in eugonadal and hypogonadal men. Fertil. Steril..

[32] Dobs A.S., Meikle A.W., Arver S., Sanders S.W., Caramelli K.E., Mazer N.A. (1999). Pharmacokinetics, efficacy, and safety of a permeation-enhanced testosterone transdermal system in comparison with bi-weekly injections of testosterone enanthate for the treatment of hypogonadal men. J. Clin. Endocrinol. Metab..

[33] Matsumoto A.M. (1990). Effects of chronic testosterone administration in normal men: Safety and efficacy of high dosage testosterone and parallel dose-dependent suppression of luteinizing hormone, follicle-stimulating hormone, and sperm production. J. Clin. Endocrinol. Metab..

[34] Kaminetsky J., Jaffe J.S., Swerdloff R.S. (2015). Pharmacokinetic Profile of Subcutaneous Testosterone Enanthate Delivered via a Novel, Prefilled Single-Use Autoinjector: A Phase II Study. Sex. Med..

[35] Yates W.R., Perry P.J., MacIndoe J., Holman T., Ellingrod V. (1999). Psychosexual effects of three doses of testosterone cycling in normal men. Biol. Psychiatry.

[36] Anderson R.A., Wu F.C. (1996). Comparison between testosterone enanthate-induced azoospermia and oligozoospermia in a male contraceptive study. II. Pharmacokinetics and pharmacodynamics of once weekly administration of testosterone enanthate. J. Clin. Endocrinol. Metab..

[37] O’Connor D.B., Archer J., Hair W.M., Wu F.C. (2002). Exogenous testosterone, aggression, and mood in eugonadal and hypogonadal men. Physiol. Behav..

[38] Tricker R., Casaburi R., Storer T.W., Clevenger B., Berman N., Shirazi A., Bhasin S. (1996). The effects of supraphysiological doses of testosterone on angry behavior in healthy eugonadal men—A clinical research center study. J. Clin. Endocrinol. Metab..

[39] Bhasin S., Storer T.W., Berman N., Callegari C., Clevenger B., Phillips J., Bunnell T.J., Tricker R., Shirazi A., Casaburi R. (1996). The effects of supraphysiologic doses of testosterone on muscle size and strength in normal men. N. Eng. J. Med..

[40] Seidman S.N., Rabkin J.G. (1998). Testosterone replacement therapy for hypogonadal men with SSRI-refractory depression. J. Affect. Disord..

[41] Bachman E., Feng R., Travison T., Li M., Olbina G., Ostland V., Ulloor J., Zhang A., Basaria S., Ganz T. (2010). Testosterone suppresses hepcidin in men: A potential mechanism for testosterone-induced erythrocytosis. J. Clin. Endocrinol. Metab..

[42] Herbst K.L., Amory J.K., Brunzell J.D., Chansky H.A., Bremner W.J. (2003). Testosterone administration to men increases hepatic lipase activity and decreases HDL and LDL size in 3 wk. Am. J. Physiol. Endocrinol. Metab..

[43] Bhasin S., Woodhouse L., Casaburi R., Singh A.B., Bhasin D., Berman N., Chen X., Yarasheski K.E., Magliano L., Dzekov C. (2001). Testosterone dose-response relationships in healthy young men. Am. J. Physiol. Endocrinol. Metab..

[44] Cantrill J.A., Dewis P., Large D.M., Newman M., Anderson D.C. (1984). Which testosterone replacement therapy?. Clin. Endocrinol..

[45] Solheim S.A., Mørkeberg J., Juul A., Freiesleben S.Y., Upners E.N., Dehnes Y., Nordsborg N.B. (2020). An Intramuscular Injection of Mixed Testosterone Esters Does Not Acutely Enhance Strength and Power in Recreationally Active Young Men. Front. Physiol..

[46] Nankin H.R. (1987). Hormone kinetics after intramuscular testosterone cypionate. Fertil. Steril..

[47] Yale University Elevated Testosterone Kills Nerve Cells. ScienceDaily.

[48] Nagelberg S.B., Laue L., Loriaux D.L., Liu L., Sherins R.J. (1986). Cerebrovascular accident associated with testosterone therapy in a 21-year-old hypogonadal man. N. Engl. J. Med..

[49] Willson C.M., Grundmann O. (2017). In vitro assays in natural products research—A matter of concentration and relevance to in vivo administration using resveratrol, α-mangostin/γ-mangostin and xanthohumol as examples. Nat. Prod. Res..

[50] Willson C. (2020). Comments to the Editor Re: Papukashvili et al. Nutrients.

[51] Lanthier A., Patwardhan V.V. (1986). Sex steroids and 5-en-3 beta-hydroxysteroids in specific regions of the human brain and cranial nerves. J. Steroid Biochem..

[52] Hammond G.L., Hirvonen J., Vihko R. (1983). Progesterone, androstenedione, testosterone, 5 alpha-dihydrotestosterone and androsterone concentrations in specific regions of the human brain. J. Steroid Biochem..

[53] Rosario E.R., Chang L., Stanczyk F.Z., Pike C.J. (2004). Age-related testosterone depletion and the development of Alzheimer disease. JAMA.

[54] Rosario E.R., Chang L., Head E.H., Stanczyk F.Z., Pike C.J. (2011). Brain levels of sex steroid hormones in men and women during normal aging and in Alzheimer’s disease. Neurobiol. Aging.

[55] Bloch M., Rubinow D.R., Berlin K., Kevala K.R., Kim H.Y., Schmidt P.J. (2006). Monoamines and neurosteroids in sexual function during induced hypogonadism in healthy men. Arch. Gen. Psychiatry.

[56] Banks W.A., Morley J.E., Niehoff M.L., Mattern C. (2009). Delivery of testosterone to the brain by intranasal administration: Comparison to intravenous testosterone. J. Drug Target..

[57] Caruso D., Melis M., Fenu G., Giatti S., Romano S., Grimoldi M., Crippa D., Marrosu M.G., Cavaletti G., Melcangi R.C. (2014). Neuroactive steroid levels in plasma and cerebrospinal fluid of male multiple sclerosis patients. J. Neurochem..

[58] Martin J., Plank E., Jungwirth B., Hapfelmeier A., Podtschaske A., Kagerbauer S.M. (2019). Weak correlations between serum and cerebrospinal fluid levels of estradiol, progesterone and testosterone in males. BMC Neurosci..

[59] Ryberg H., Johansson P., Wallin A., Emilsson J.F., Eriksson E., Svensson J., Ohlsson C. (2022). Testosterone associates differently with body mass index and age in serum and cerebrospinal fluid in men. J. Intern. Med..

[60] Dubey A.K., Herbert J., Abbott D.H., Martensz N.D. (1984). Serum and CSF concentrations of testosterone and LH related to negative feedback in male rhesus monkeys. Neuroendocrinology.

[61] Kancheva R., Hill M., Novák Z., Chrastina J., Velíková M., Kancheva L., Ríha I., Stárka L. (2010). Peripheral neuroactive steroids may be as good as the steroids in the cerebrospinal fluid for the diagnostics of CNS disturbances. J. Steroid Biochem. Mol. Biol..

[62] Daly R.C., Su T.P., Schmidt P.J., Pickar D., Murphy D.L., Rubinow D.R. (2001). Cerebrospinal fluid and behavioral changes after methyltestosterone administration: Preliminary findings. Arch. Gen. Psychiatry.

[63] Cunningham G.R., Tindall D.J., Lobl T.J., Campbell J.A., Means A.R. (1981). Steroid structural requirements for high affinity binding to human sex steroid binding protein (SBP). Steroids.

[64] Wiita B., Artis A., Ackerman D.M., Longcope C. (1995). Binding of 17-alpha-methyltestosterone in vitro to human sex hormone binding globulin and rat ventral prostate androgen receptors. Ther. Drug Monit..

[65] Teubel J., Parr M.K. (2020). Determination of neurosteroids in human cerebrospinal fluid in the 21st century: A review. J. Steroid Biochem. Mol. Biol..

[66] Kasteel E.E.J., Westerink R.H.S. (2021). Refining in vitro and in silico neurotoxicity approaches by accounting for interspecies and interindividual differences in toxicodynamics. Expert Opin. Drug Metab. Toxicol..

[67] Minta M., Radko L., Stypuła-Trębas S., Żmudzki J. (2014). Cytotoxic effects of the synthetic oestrogens and androgens on Balb/c 3T3 and HepG2 cells. Bull. Vet. Inst. Pulawy.

[68] Bal-Price A.K., Hogberg H.T., Buzanska L., Coecke S. (2010). Relevance of in vitro neurotoxicity testing for regulatory requirements: Challenges to be considered. Neurotoxicol. Teratol..

[69] Heusinkveld H.J., Westerink R.H.S. (2017). Comparison of different in vitro cell models for the assessment of pesticide-induced dopaminergic neurotoxicity. Toxicol. Vitr..

[70] Gao L., Zhou W., Symmes B., Freed C.R. (2016). Re-Cloning the N27 Dopamine Cell Line to Improve a Cell Culture Model of Parkinson’s Disease. PLoS ONE.

[71] Su C., Rybalchenko N., Schreihofer D.A., Singh M., Abbassi B., Cunningham R.L. (2012). Cell Models for the Study of Sex Steroid Hormone Neurobiology. J. Steroids Horm. Sci..

[72] Bjørnebekk A., Walhovd K.B., Jørstad M.L., Due-Tønnessen P., Hullstein I.R., Fjell A.M. (2017). Structural Brain Imaging of Long-Term Anabolic-Androgenic Steroid Users and Nonusing Weightlifters. Biol. Psychiatry.

[73] Bjørnebekk A., Kaufmann T., Hauger L.E., Klonteig S., Hullstein I.R., Westlye L.T. (2021). Long-term Anabolic-Androgenic Steroid Use Is Associated with Deviant Brain Aging. Biol. Psychiatry Cogn. Neurosci. Neuroimaging.

[74] Albano G.D., Amico F., Cocimano G., Liberto A., Maglietta F., Esposito M., Rosi G.L., Di Nunno N., Salerno M., Montana A. (2021). Adverse Effects of Anabolic-Androgenic Steroids: A Literature Review. Healthcare.

[75] Hauger L.E., Westlye L.T., Fjell A.M., Walhovd K.B., Bjørnebekk A. (2019). Structural brain characteristics of anabolic-androgenic steroid dependence in men. Addiction.

[76] Archer E., Pavela G., Lavie C.J. (2015). The Inadmissibility of What We Eat in America and NHANES Dietary Data in Nutrition and Obesity Research and the Scientific Formulation of National Dietary Guidelines. Mayo Clin. Proc..

[77] Hauger L.E., Havnes I.A., Jørstad M.L., Bjørnebekk A. (2021). Anabolic androgenic steroids, antisocial personality traits, aggression and violence. Drug Alcohol Depend..

[78] Vaskinn A., Hauger L.E., Bjørnebekk A. (2020). Theory of mind in users of anabolic androgenic steroids. Psychopharmacology.

[79] Nelson B.S., Hildebrandt T., Wallisch P. (2022). Anabolic-androgenic steroid use is associated with psychopathy, risk-taking, anger, and physical problems. Sci. Rep..

[80] Ersche K.D., Turton A.J., Chamberlain S.R., Müller U., Bullmore E.T., Robbins T.W. (2012). Cognitive dysfunction and anxious-impulsive personality traits are endophenotypes for drug dependence. Am. J. Psychiatry.

[81] Mackey S., Allgaier N., Chaarani B., Spechler P., Orr C., Bunn J., Allen N.B., Alia-Klein N., Batalla A., Blaine S. (2019). Mega-Analysis of Gray Matter Volume in Substance Dependence: General and Substance-Specific Regional Effects. Am. J. Psychiatry.

[82] MacPhail D.C., Oberle C.D. (2022). Seeing Shred: Differences in muscle dysmorphia, orthorexia nervosa, depression, and obsessive-compulsive tendencies among groups of weightlifting athletes. Perform. Enhanc. Health.

[83] Garcia-Argibay M. (2019). The Relationship between the Big Five Personality Traits, Impulsivity, and Anabolic Steroid Use. Subst. Use Misuse.

[84] Cafri G., Olivardia R., Thompson J.K. (2008). Symptom characteristics and psychiatric comorbidity among males with muscle dysmorphia. Compr. Psychiatry.

[85] Greenway C.W., Price C. (2018). A qualitative study of the motivations for anabolic-androgenic steroid use: The role of muscle dysmorphia and self-esteem in long-term users. Perform. Enhanc. Health.

[86] Longobardi C., Prino L.E., Fabris M.A., Settanni M. (2017). Muscle dysmorphia and psychopathology: Findings from an Italian sample of male bodybuilders. Psychiatry Res..

[87] Daley M.M., Reardon C.L. (2021). Bipolar Disorder and Athletes: A Narrative Review. Curr. Sports Med. Rep..

[88] Sagoe D., McVeigh J., Bjørnebekk A., Essilfie M.S., Andreassen C.S., Pallesen S. (2015). Polypharmacy among anabolic-androgenic steroid users: A descriptive metasynthesis. Subst. Abuse Treat. Prev. Policy.

[89] Harvey O., Keen S., Parrish M., van Teijlingen E. (2019). Support for people who use Anabolic Androgenic Steroids: A Systematic Scoping Review into what they want and what they access. BMC Public Health.

[90] Wilson J.D. (1988). Androgen abuse by athletes. Endocr. Rev..

[91] Nutt D., King L.A., Saulsbury W., Blakemore C. (2007). Development of a rational scale to assess the harm of drugs of potential misuse. Lancet.

[92] van Amsterdam J., Opperhuizen A., Hartgens F. (2010). Adverse health effects of anabolic-androgenic steroids. Regul. Toxicol. Pharmacol..

[93] Hoffman J.R., Ratamess N.A. (2006). Medical issues associated with anabolic steroid use: Are they exaggerated?. J. Sports Sci. Med..

[94] Street C., Antonio J., Cudlipp D. (1996). Androgen use by athletes: A reevaluation of the health risks. Can. J. Appl. Physiol..

[95] Smit D.L., de Ronde W. (2018). Outpatient clinic for users of anabolic androgenic steroids: An overview. Neth. J. Med..

[96] Smit D.L., Bond P., de Ronde W. (2022). Health effects of androgen abuse: A review of the HAARLEM study. Curr. Opin. Endocrinol. Diabetes Obes..

[97] Alger M., Luera N.A., Weiner R. (2022). What are the benefits and harms of testosterone replacement therapy in men with age-related low testosterone?. Evid.-Based Pract..

